# Competing microorganisms with exclusion effects against multidrug-resistant *Salmonella* Infantis in chicken litter supplemented with growth-promoting antimicrobials

**DOI:** 10.14202/vetworld.2025.1127-1136

**Published:** 2025-05-13

**Authors:** María Alejandra Ospina Barrero, Maryeimy Varón-López, Lina M. Peñuela-Sierra

**Affiliations:** 1 Laboratory of Microbiology and Mycorrhiza, Research Group on Plant and Microbial Biotechnology - GEBIUT, Faculty of Sciences, University of Tolima, Ibagué, Colombia; 2 Veterinary Medicine and Animal Science Program, Universidad Cooperativa de Colombia, Ibagué 730003, Tolima, Colombia; 3 Department of Veterinary Medicine and Animal Science, Faculty of Veterinary Medicine and Animal Science, University of Tolima, Ibagué, Colombia

**Keywords:** antimicrobial resistance, *Bacillus subtilis*, competitive exclusion, food safety, *Lactobacillus plantarum*, poultry litter, *Salmonella* Infantis

## Abstract

**Background and Aim:**

The widespread use of antibiotic growth promoters (AGPs) in poultry production has been implicated in altering gut microbiota and promoting the excretion of multidrug-resistant (MDR) bacteria into the environment. *Salmonella enterica* serovar Infantis (*Salmonella* Infantis [S.I]), a prevalent zoonotic pathogen, has demonstrated increasing resistance in poultry systems. This study aimed to evaluate the efficacy of natural control microorganisms (NCM), *Bacillus subtilis* and *Lactobacillus plantarum*, in reducing the abundance of MDR S.I in fresh chicken litter from birds raised with or without AGP supplementation. It also examined how physicochemical properties and microbial dynamics influence pathogen persistence.

**Materials and Methods:**

Microcosms were constructed using litter from broilers raised under two dietary regimes (with and without avilamycin). Treatments included combinations of AGP, S.I, and NCM. Bacterial enumeration was performed using selective media, and whole-genome sequencing of S.I was conducted to characterize antimicrobial resistance and virulence genes. Physicochemical parameters (pH, humidity, temperature, and ammonia) were measured and correlated with microbial loads. Antagonistic activity of NCM strains was assessed using agar diffusion assays.

**Results:**

Genome analysis revealed that S.I carried multiple resistance genes (e.g., *bla*CTX-M-*65*, *tet(A)*, and *sul1*) and efflux systems conferring MDR. *In vitro* assays showed strong antagonism by *L. plantarum* and moderate activity by *B. subtilis*. In microcosms, S.I counts significantly decreased in the presence of both AGP and NCM, indicating synergistic inhibition. Conversely, in the absence of AGP, NCM had a limited effect. Statistical analyses showed strong correlations between microbial groups and physicochemical variables, particularly during later production stages.

**Conclusion:**

The application of *B. subtilis* and *L. plantarum* in chicken litter significantly reduced S.I colonization under AGP supplementation, suggesting their potential as biocontrol agents. These findings support the development of integrated litter management strategies to mitigate zoonotic and resistant pathogen dissemination, particularly in AGP-using systems. However, the effectiveness of such interventions may vary across farms due to differences in microbial ecology and environmental conditions.

## INTRODUCTION

The poultry industry has emerged as the lea-ding global provider of efficient, high-quality animal protein [1], resulting in the intensification of production systems that generate substantial volumes of waste, including the litter used for housing chickens. Chicken litter is composed of wood shavings, rice husks, or saw-dust mixed with feed residues and chicken excreta [2]. Concurrently, the increased demand for chicken meat has been accompanied by the indiscriminate use of antimicrobial agents. These agents are employed both to prevent or treat infectious diseases and as antibiotic growth promoters (AGPs), which modulate the intestinal microbiota, enhance nutrient absorption, and improve growth performance [3, 4]. However, these substances are not fully metabolized by the birds, leading to their excretion in feces and accumulation in litter, soil, and wastewater. This accumulation alters microbial ecosystems by eliminating susceptible bacterial strains while promoting the survival of resistant ones [4, 5].

*Salmonella* spp., particularly *Salmonella enterica* serovar Infantis (*Salmonella* Infantis [S.I]), is among the most widely distributed zoonotic pathogens in poultry litter globally. It is frequently isolated in regions such as Europe, the United States, and Latin America, with an estimated flock-level prevalence of 9% [6]. Although often asymptomatic in commercial poultry [7], S.I can result in elevated mortality rates and reduced productivity in broilers, depending on flock management and immune status [8]. Moreover, S.I is a significant cause of foodborne outbreaks and is resistant to multiple antibiotics, thereby compromising treatment efficacy and increasing the risk of severe human illness [8]. This strain exhibits resistance to several antimicrobial classes, notably quinolones, tetracyclines, and sulfonamides [9].

Infected birds shed S.I through feces, and its environmental persistence is influenced by factors such as serotype, temperature, moisture content, pH, and the physical or chemical treatment of litter before reuse [8, 10]. Consequently, European Union regulations mandate the removal and replacement of litter at the end of each production cycle to minimize contamination risk [11, 12]. Nevertheless, due to the high cost of fresh litter and efforts to reduce on-farm waste, many producers opt to recycle litter across multiple flocks for a year or more [2]. To mitigate pathogen loads in reused litter, chemical, physical, and biological interventions have been explored, including the application of natural control microorganisms (NCM) (e.g., *Bacillus subtilis*) [13, 14]. For instance, broiler litter treated with a commercial probiotic-based product – comprising an eco-friendly detergent and spores of *B. subtilis*, *Bacillus pumilus*, and *Bacillus megaterium* at a concentration of 5 × 10^8^ colony-forming units (CFU)/mL (Chrisal, Lommel, and Belgium) – demonstrated reduced counts of total aerobic bacteria, *Enterobacteriaceae*, and coagulase-positive *Staphylococci* [13]. Competitive exclusion is considered a highly effective approach to prevent *Salmonella* colonization in broilers and underpins the development of probiotics, microbial consortia, biocontrol agents, and cleaning solutions used extensively in animal husbandry [13].

Exposure of birds to litter enriched with enteric and environmental microorganisms supports early microbiota development and intestinal colonization by diverse bacterial populations. These communities inhibit *Salmonella* invasion through mechanisms such as competitive exclusion at adherence sites, compe-tition for nutrients, production of short-chain volatile fatty acids, and secretion of antimicrobial peptides (bacteriocins) by lactic acid bacteria – including *Lacto-bacillus*, *Pediococcus*, *Lactococcus*, *Enterococcus*, and *Streptococcus* [13, 14].

Despite growing concerns over the proliferation of MDR *S. enteric*a S.I in poultry production systems, particularly in reused chicken litter, there remains limited understanding of how NCM, such as *B. subtilis* and *Lactobacillus plantaru*m, interact with the micro-biota and physicochemical environment of poultry litter under AGP supplementation. While prior studies have demonstrated the *in vitr*o antagonistic potential of these NCMs and their role in competitive exclu-sion, their efficacy *in vivo* – particularly in litter derived from poultry supplemented with AGPs – remains insufficiently characterized. Moreover, the influence of litter physicochemical properties on pathogen dynamics and microbial community interactions in the context of NCM inoculation is poorly understood. These knowledge gaps hinder the development of sustainable litter management strategies aimed at mitigating zoonotic risk and controlling MDR pathogens in poultry systems.

This study aims to evaluate the exclusionary potential of *B. subtilis* and *L. plantarum* against MDR S.I in fresh chicken litter derived from broilers reared with and without AV supplementation. Specifically, the research investigates: (i) the antagonistic activity of these NCMs under controlled microcosm conditions; (ii) the modulation of S.I abundance in response to AGP and NCM treatments; (iii) the genomic characteristics and antimicrobial resistance profile of the isolated S.I strain; and (iv) the relationship between physicochemical litter parameters and microbial community dynamics. The findings are expected to inform evidence-based strategies for microbiological safety enhancement in poultry litter and contribute to antimicrobial resistance mitigation in animal agriculture.

## MATERIALS AND METHODS

### Ethical approval

Ethical approval was not required for this study, as chicken litter samples were collected without dir-ect contact with the birds. Furthermore, bacterial inoculation was performed exclusively on the bedding material used in the construction of the microcosms. All samples were collected aseptically in accordance with established collection protocols.

### Study period and location

The study was conducted from March 2022 to December 2022 at Brisas Farm and the Microbiology and Mycorrhiza Laboratory (LMM), University of Tolima, Ibagué, Colombia.

### Detection and characterization of S.I

The S.I strain used in the microcosm infection treatments (described in the microcosm assembly section) was isolated from broiler chicken litter at a poultry farm in Tolima, Colombia, by the GEBIUT research group at the University of Tolima. The strain was characterized biochemically, phenotypically, and molecularly using 16S rRNA sequencing [11].

### Whole-genome sequencing and analysis

Genomic DNA from the S.I isolate was extracted using the boiling method [15]. DNA concentration and quality were assessed using a NanoDrop 2000c spectrophotometer (Thermo Fisher Scientific, Wilmington, DE, USA) and a Qubit fluorometer (Invitrogen, Carlsbad, CA, USA) with a Qubit dsDNA HS kit (Thermo Fisher Scientific, USA).

Whole-genome sequencing was conducted by Macrogen, Inc. (Seoul, Republic of Korea). A sequencing library was prepared using the TruSeq DNA Nano kit (Illumina, NE, USA) according to the manufacturer’s instructions, and sequencing was performed on the Illumina NovaSeq platform (150PE), generating paired-end reads. Raw data quality was assessed using FastQC [16], evaluating base composition, G+C content, sequence length distribution, and sequence duplication to identify contaminants and adapters. Low-quality reads (below Q20) were removed using Trimmomatic (v. 0.039) [17]. The filtered reads were assembled with MaSuRCA v4.1.0 [18], and the assembly was evaluated using QUAST v5.0.2 [19].

Subsequent genome annotation was carried out using Prokka v1.14.6 (Genome Annotation Soft-ware) [20], enabling identification of genomic feat-ures and contig coordinates. Species identification was performed by comparing the assembled genome to 11 reference genomes (Annex 1), selected based on the phenotypic and serological characteristics of the isolate. Negative controls – *Salmonella bongori* N268-08 and *Escherichia coli* K-12 MG1655 – yielded low identity values (89.9% and 80.6%, respectively). Antimicrobial resistance genes and virulence factors were analyzed using ResFinder 4.0 [21] and PathoFact [22]. The genome sequence was deposited in the NCBI GenBank database under accession number SUB15012476; BioProject ID: PRJNA1217447.

### Antimicrobial susceptibility analysis

Antimicrobial susceptibility testing was per-formed using the Kirby–Bauer disk diffusion method in accordance with Clinical and Laboratory Standards Institute (CLSI, 2017) guidelines [23]. The isolate was cultured on trypticase soy agar (TSA, Oxoid, Basing-stoke, UK) and incubated at 37°C overnight. Bacterial suspensions were adjusted to an optical density of 0.08 at 600 nm using a MAPADA spectrophotometer (Shanghai Mapada Instruments Co., Ltd., China) and inoculated onto Mueller–Hinton agar plates (BD Difco). *Salmonella* Typhimurium ATCC 14028 was used as the quality control strain. The susceptibility of the S.I isolate was evaluated against 15 antimicrobial agents: ampicillin/sulbactam (10 μg), amoxicillin (10 μg), gentamicin (10 μg), ciprofloxacin (10 μg), cefotaxime (30 μg), erythromycin (15 μg), nalidixic acid (30 μg), penicillin (10 μg), trimethoprim-sulfamethoxazole (25 μg), tetracycline (30 μg), ceftiofur (30 μg), enrofloxacin (5 μg), colistin (10 μg), streptomycin (10 μg), and doxycycline (30 μg).

### *In vitro* antagonistic activity of *B. subtilis* and *L. plantarum* against S.I

The *B. subtilis* ATCC 6633® and *L. plantarum* ATCC 8014® strains, known for their antagonistic activity and frequent isolation from poultry litter, were evaluated for their ability to inhibit S.I. The agar diffusion method described by Balouiri *et al*. [24] was employed. Each strain was tested in triplicate at a concentration of 10^8^ CFU/mL. The inhibition zones were measured in millimeters, and strains showing a distinct zone were considered positive for antagonistic activity [25]. Strains were preserved at −80°C in 30% glycerol (Sigma, St. Louis, MO). S.I and *B. subtilis* were cultured in Brain Heart Infusion (BHI, Oxoid®) at 37°C for 24 h, and *L. plantarum* in MRS broth (Difco^TM^, USA) at 34°C for 48 h [26].

### Experimental design

The study was conducted at Brisas Farm and the Mycorrhiza Laboratory, University of Tolima, Ibagué, Colombia (1285 m above sea level; average temperature: 26°C). Forty Ross 308 broilers were randomly assigned to two experimental groups (20 birds per group). Broiler starter and finisher diets were formulated with two levels of AV (0 and 10 g/ton). Birds were reared on fresh rice husk litter (10 cm depth) under commercial-like conditions for 42 days. Samples were collected on days 1, 7, 21, and 42. Composite litter samples were obtained from 10 points per pen, stored in sterile Nazco (USA) bags under refrigeration, and transported immediately to the laboratory for microcosm setup under biosafety conditions.

### Microcosm assembly

*Salmonella*-free status of the facilities, birds, and litter was confirmed through bacteriological culture and biochemical tests. The moisture content of samples was assessed to ensure the inoculum volume yielded 27% ± 0.1% moisture (water activity 0.90 ± 0.02).

In sterile plastic containers, 240 g of litter was placed to a depth of 10 cm, replicating shed conditions. Six treatments were tested (four replicates each): T0 (chicken litter, CL), T1 (CL + AV), T2 (CL + S.I), T3 (CL + AV + S.I), T4 (CL + *B. subtilis* + *L. plantarum* + S.I), and T5 (CL + AV + NCM + S.I) (Table 1). Each treatment was inoculated with 10^8^ CFU of the respective microorganisms and incubated in a sterile room at 26°C for 48 h. Growth kinetics of S.I, *B. subtilis*, and *L. plantarum* were monitored through absorbance at 625 nm and viable cell counts at 12, 24, and 48 h [26].

**Table 1:** Denomination of phases and treatments in the microcosms.

Starter phase: New litter–1 day old chickens	T0: CL	T1: CL+AGP	T2: CL+S.I.	T4: CL+S.I.+NCM	T5: CL+AGP+S.I.+NCM
Growing I phase: 7 days chickens	T0: CL	T1: CL+AGP	T2: CL+S.I.	T4: CL+S.I.+NCM	T5: CL+AGP+S.I.+NCM
Growing II Phase–21 days old chickens	T0: CL	T1: CL+AGP	T2: CL+S.I.	T4: CL+S.I.+NCM	T5: CL+AGP+S.I.+NCM
Finisher phase 42: Chickens	T0: CL	T1: CL+AGP	T2: CL+S.I.	T4: CL+S.I.+NCM	T5: CL+AGP+S.I.+NCM

CL=Chicken litter, AGP=Avilamycin growth promoter antimicrobial, S.I=*S* Infantis, NCM=*Bacillus subtilis* and *Lactobacillus plantarum*

### Chicken litter sampling in microcosms

To enumerate CFU, 10 g of litter was homogenized with 90 mL of buffered peptone water (BPW, Oxoid Ltd., Ogdensburg, NY) using a Stomacher mixer (Bag Mixer® 400, Interscience Co., France) for 1 min. Decimal dilutions up to 10^−4^ were plated in triplicate. Aerobic mesophilic bacteria were counted using Plate Count Agar (Oxoid, Milan, Italy) following ISO 4833-1 [27]. *Enterobacteriaceae* were enumerated on Violet Red Bile Glucose Agar per ISO 21528-2:2017 [28]. S.I was enumerated on XLT4 agar following ISO 6579-1:2017 [29]. *Bacillus* and *Lactobacillus* counts were determined using Luria Bertani and MRS media, respectively. For each replicate, 100 g of litter was collected to measure temperature, moisture, pH, and ammonia concentration.

### Statistical analysis

Microbial counts were log-transformed (log CFU g^−1^) to compute means and standard deviations across treatments. The Kruskal–Wallis test was applied due to non-normal data distribution, to determine significant differences among treatments and production stages. Data visualization was conducted using box-and-whisker plots. Dunn’s *post hoc* test and Spearman correlation were employed to explore relationships between microbial abundance and physicochemical parameters, and to confirm correlations with *Salmonella* presence.

## RESULTS

### Genetic characteristics of S.I

To elucidate the genetic features contributing to the survival of S.I in chicken litter under various treatments, whole-genome sequencing was conducted. The isolate exhibited 99.83% nucleotide identity with *S. enterica* subsp. *enterica* S.I 1326/28 (United Kingdom), with a genome size of approximately 5.5 Mbp. A total of 2,662 scaffolds were assembled, with an average GC content of 52.2% and a total length of 5,555,760 base pairs.

The pangenome analysis identified 4,414 genes, including 3,353 core genes essential for basic cellular processes such as DNA replication, protein synthesis, and metabolism, totaling 7,767 genes. Nine genes located on either the chromosomes or plasmids conferred resistance to six antimicrobial classes, including amino-glycosides, β-lactams, phenicols, sulfonamides, and tetracyclines (Table 2). In some cases, the genetic determinants correlated with phenotypic resistance, including genes conferring resistance to cefotaxime, gentamicin, tetracycline, and streptomycin.

**Table 2:** Antimicrobial resistance genes of *Salmonella* Infantis.

AMR gene	Genetic background	Class of antimicrobials conferred by resistance	Antimicrobial agent conferred resistance
*aac(6’)-Iaa*	NC_003197	Aminoglycosides	Amikacin, tobramycin
*aadA1*	JX185132		Spectinomycin and streptomycin
*aadA1*	JQ414041		Spectinomycin and streptomycin
*aph(4)-Ia*	V01499		Hygromycin
aac(3)-IV	DQ241380		Gentamicin and tobramycin
*bla*CTX-M65	EF418608	β-lactams	Amoxicillin, ampicillin, azithromycin, cefepime, cefotaxime, ceftazidime, ceftriaxone, piperacillin, ticarcillin
*floR*	AF118107	Fenicoles	Chloramphenicol, florfenicol
*sul1*	U12338	Sulfonamides	Sulfamethoxazole
*tet(A)*	AJ517790	Tetracyclines	Doxycycline; tetracycline

Multiple drug efflux systems were also identified. These included the major facilitator superfamily (MFS), with pumps such as MdtK, EmrB, and MdfA, which expel quinolones. MdfA additionally conferred resistance to tetracycline, chloramphenicol, and norfloxacin [30, 31]. The resistance-nodulation-division (RND) efflux syst-ems, responsible for eliminating quinolones, amino-glycosides, sulfonamides, chloramphenicol, macrolides, and tetracycline, were also detected [32]. The small multidrug resistance (SMR) family, associated with the transport of quaternary ammonium compounds found in disinfectants and antiseptics, and the ATP-binding cassette (ABC) transporters, involved in virulence, host-pathogen interactions, nutrient uptake, and macrolide efflux, were also present [33].

In total, 3,278 virulence genes were identified, including those associated with biofilm formation, pathogenicity islands, and effector proteins. Notable examples include: *sseL*, involved in macrophage suppr-ession during infection; *sipA*, *sipD*, and *sopD*, which regulate actin cytoskeleton rearrangement; *csgA*, encoding curli fimbriae; *invA*, essential for invasion; *sifA*, involved in intracellular survival; *pipB2*, which modulates host responses; *csgD*, involved in biofilm regulation; siderophore-related genes (*entA*–*entF*); *fepG*, for ferric ion transport; and stress response regulators such as *PhoP*, *PhoQ*, and *RpoS* [34–37].

### Antimicrobial Susceptibility of S.I

Antimicrobial susceptibility testing using the disk diffusion method revealed that the S.I isolate exhibited an MDR phenotype. Resistance was observed to sev-eral antimicrobial classes, including cefotaxime, genta-micin, nalidixic acid, penicillin, tetracycline, ceftiofur, and streptomycin. The isolate showed intermediate susce-ptibility to ciprofloxacin and was resistant to all other tested agents (Table 3).

**Table 3:** Phenotypic resistance of *Salmonella* Infantis

Antimicrobial	Concentration (µg)	Inhibition zone diameter (mm)			*Salmonella* Infantis		
	
		S	I	R	S	I	R
Cefotaxima	30 µg	≥8	16-32	≤64			+
Ciprofloxacin	10 µg	≥31	21-30	≤20		+	
Gentamicin	10 µg	≥4	8	≤16			+
Erythromycin	15 µg	≥23	14-22	≤ 13			+
Nalidixic acid	30 µg	>19	14-18	<13			+
Penicillin	10 µg	≥22	12-21	≤11			+
Tetracycline	30 µg	>15	12-14	<11			+
Ceftiofur	30 µg	≥21	18-20	≤17			+
Streptomycin	10 µg	>15	12-14	≤11			+

S: Sensitive, I: Intermediate sensitivity, and R: Resistant.

### *In vitro* antagonistic activity of *B. subtilis* and *L. plantarum*

According to the criteria established by Abdel-Daim *et al*. [38] (strong inhibition: ≥15 mm; moderate: 10–15 mm; weak: ≤10 mm; resistant: 0 mm), *B. subtilis* exhibited moderate antagonistic activity against S.I, while *L. plantarum* demonstrated strong inhibitory effects.

### Effects of treatments on S.I in chicken litter microcosms

To assess the impact of NCM inoculation on S.I dynamics in chicken litter under AGP-supplemented and non-supplemented conditions, initial verification confirmed the absence of *Salmonella* and the presence of *Bacillus* and *Lactobacillus* in all samples.

**Figure 1:**
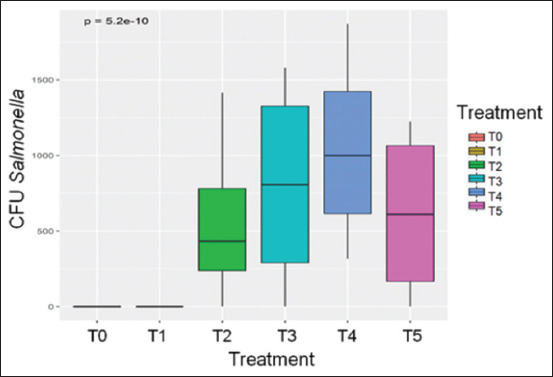
Effect of treatments on CFU of *Salmonella* Infantis in chicken litter. CFU=Colony-forming units

The lowest S.I CFU was observed in the treatment supplemented with both AV and NCM (T5), followed by treatments without supplementation (T2), with AV alone (T3), and with NCM alone (T4). A significant reduction (p < 0.05) in CFU was evident between T5 and T4, indicating that *B. subtilis* and *L. plantarum* are more effective at reducing *Salmonella* populations when combined with AV. This conclusion is further supported by the greater reduction in S.I in T5 compared to T3. In addition, results suggest that indigenous microbial communities in non-supplemented litter contribute to the regulation of *Salmonella* proliferation (Figure 1).

### Dynamics of S.I during the production cycle

**Figure 2:**
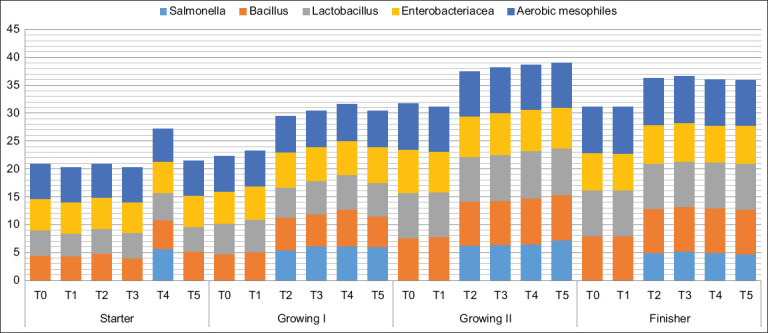
Relative abundance of *Salmonella, Bacillus, Enterobacteriacea*, and *Aerobic mesophiles* cultivable in litter during the broiler production. Starter phase: New litter – 1 day old chickens, Growing I phase: 7 days old chickens, Growing II phase: 21 days old chickens, Finisher phase: 42 days chickens.

Figure 2 presents the culturable bacterial community profiles across different production stages. *Salmonella* was not detected in T0 or T1, indicating no contamination from farm or experimental sources. In contrast, S.I persisted throughout the cycle in T4. During the initial stage, only T4 showed *Salmonella* presence. In the rearing and fattening stages, the relative abundance of bacterial communities increased, with S.I detected in T2, T3, T4, and T5. The fattening stage displayed the highest overall microbial diversity. Notably, all microbial groups, including *Salmonella*, declined by the end of the production cycle

### Influence of physicochemical parameters on S.I abundance

**Figure 3:**
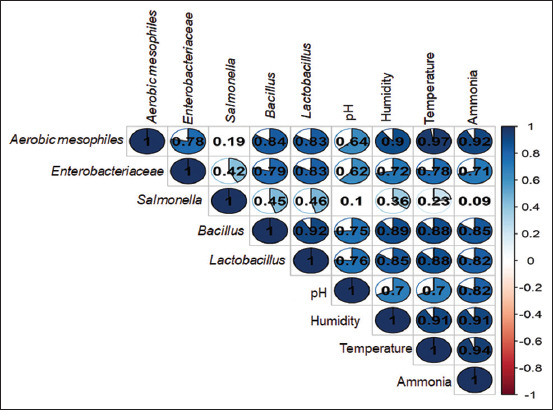
Correlation of *Aerobic mesophiles*, *Enterobacteriaceae*, *Salmonella, Bacillus*, and *Lactobacillus* with the physicochemical properties of chicken litter.

Figure 3 illustrates the relationships between microbial groups and physicochemical parameters of the litter. Strong positive correlations (r > 0.8) were observed between aerobic mesophiles and *Enterobacteriaceae* with *Bacillus*, *Lactobacillus*, humidity, temperature, and ammonia concentration. *Salmonella* showed a weaker positive correlation (r ≈ 0.4) with *Bacillus*, *Lactobacillus*, humidity, and temperature. Interestingly, during the finishing stage – when *Salmonella* viability decreased – the abundances of *Bacillus* and *Lactobacillus* increased.

The presence of *Bacillus* and *Lactobacillus* was strongly correlated (r > 0.7) with pH, humidity, temper-ature, and ammonia levels. During the production cycle, these physicochemical parameters varied as follows: pH (5.9–7.4), relative humidity (27%–36%), temperature (28°C–32°C), and ammonia concentration (0–100 ppm).

## DISCUSSION

In recent years, S.I has been classified as an emerging pathogen due to its persistence in feed environments, its high prevalence in human infections, and its resistance to multiple antimicrobial agents – traits closely linked to the widespread use of antimicrobials in food animal production systems [8, 39]. In poultry farms where S.I was isolated from treatment litter, anti-biotics were employed for prophylactic, therapeutic, and growth-promoting purposes. This practice likely contributed to the phenotypic resistance of S.I to nine antimicrobials of significance in both veterinary and human medicine. Furthermore, the selective pressure exerted by antibiotic use may explain the presence of several resistance genes, including *bla*CTX-M-65, which is associated with the persistence of *Salmonella* under beta-lactam exposure and the overexpression of efflux pumps – mechanisms contributing to the multidrug resistance profile of the isolate [40].

Therefore, identifying effective strategies to reduce pathogen loads in poultry litter is essential for enhancing feed safety and minimizing the environmental dissemination of antimicrobial resistance. Given that litter influences the gut microbiota of poultry and that the presence of zoonotic pathogens like *Salmonella* in litter before slaughter increases the likelihood of carcass contamination [41, 42], the use of litter as fertilizer also raises concerns due to the potential spread of environmentally persistent pathogens [43].

In poultry operations, litter may be freshly applied or reused multiple times, depending on material cost and availability [2, 10]. However, European regulations for broiler welfare mandate the replacement of litter between flocks [12]. Chickens raised on reused litter tend to develop a more diverse gut microbiota and exhibit stronger immune responses compared to birds raised on new litter; reuse has been associated with reduced *Salmonella* prevalence relative to first-use litter [41, 44]. Consequently, it is crucial to explore alternatives that ensure the microbiological safety of newly applied litter.

In the present study, the efficacy of *B. subtilis* and *L. plantarum* inoculation in newly applied litter for controlling MDR S.I was evaluated in microcosms during the broiler production cycle under conditions with and without AV supplementation. The most substantial reduction in S.I counts occurred in the NCM-inoculated litter of chickens supplemented with AV. This effect may be attributed to the antibacterial activity of AV against gram-positive bacteria, such as *Enterococcus*, *Streptococcus*, and *Staphylococcus* [45, 46], considering that S.I exhibited a strong positive correlation with *Staphylococcus* (data not shown). The reduction in microbial competitors due to AV treatment may have facilitated the establishment of *Bacillus* and *Lactobacillus*, which, through competitive exclusion and production of antimicrobials and bacteriocins, inhibited *Salmonella* proliferation [47]. This dual mechanism – combining AV and NCM action – holds significant potential for minimizing the development of antimicrobial resistance [48].

For litter samples without AV supplementation, the best-performing treatment was the one without NCM inoculation (T2). These findings contrast with those reported by Roll *et al.*, 2008 [49], who observed a significant reduction in *Enterobacteriaceae* in wood shavings-based litter treated with 5 g/m^2^ of Impact P® (Schering-Plough, Kenilworth, NJ, USA), a product containing *B. subtilis* and its protease enzymes. It is worth noting that the concentration of probiotic products significantly affects their efficacy, as a dose of 2.5 g/m^2^ was found ineffective. The lack of effect observed in T4 may be due to the limited concentration (10^8^ CFU) of *B. subtilis* and *L. plantarum* used in this study. Similarly, the current findings differ from those of De Cesare *et al.*, 2019 [13], who demonstrated reductions in *Enterobacteriaceae*, including *S. enterica* and *E. coli*, using a spore-based cleaning product with a higher concentration (9 × 10^9^ CFU/m²) of *B. subtilis*, *B. pumilus*, and *B. megaterium*.

The modest antagonistic effect observed for the NCM in the absence of AV (T4) may be attributed to the limited *B. subtilis* concentration and the use of a single strain. Previous studies by Shafi *et al*. [50] and Mukherjee *et al*. [51] have shown that combining *Bacillus* species with different antimicrobial mechanisms enhances efficacy against pathogens. For *L. plantarum*, the reduced performance may reflect the low nutrient content of new litter, despite its strong *in vitro* antagonistic activity. Merino *et al.*, [52] reported that *Lactobacillus* interferes with *Salmonella* biofilm formation under optimal *in vitro* conditions. However, under litter conditions with limited nutrients and suboptimal temperatures, its growth and effectiveness are reduced. Lactic acid bacteria produce a variety of antimicrobial compounds – such as organic acids, ethanol, diacetyl, and hydrogen peroxide – that lower pH, inhibit pathogen growth, and promote *Salmonella* inactivation [53].

The lowest CFU counts of S.I, regardless of treatment, were recorded during the finishing phase. This stage coincided with a more diverse gut microbiota in broilers, which was likely transferred to the litter through feces [44, 54], thereby impeding *Salmonella* survival. The successful establishment of an invading microbial species often depends on the structure of the resident community; generally, ecosystems with reduced species diversity are more vulnerable to colonization due to the availability of unoccupied ecological niches [55]. This observation is relevant because the microbial profile of litter at the finishing stage reflects both the contamination risk at slaughter and the microbiological quality of material reused in future production cycles or applied as fertilizer.

Physicochemical factors also play a critical role in microbial inactivation, and their interactions with the poultry litter microbiota are frequently studied [56]. In this study, strong positive correlations were observed between aerobic mesophiles and *Enterobacteriaceae* with environmental parameters including humidity, temperature, ammonia concentration, and pH. These variables likely promoted the proliferation of beneficial microbial groups capable of excluding *Salmonella*, particularly during the finishing phase, when such interactions are most dynamic [57]. In contrast, the survival of S.I appeared less affected by these environmental factors due to the presence of genetic adaptations, including *RpoS* (which supports survival under stress conditions such as low pH, high osmolarity, and nutrient limitation) and *MgtA* (which facilitates magnesium uptake for survival and replication) [34–37].

## CONCLUSION

This study demonstrated that the inoculation of *B. subtilis* and *L. plantarum* (NCM) into fresh chicken litter significantly reduced the abundance of MDR S.I, particularly when administered in combination with the AGP AV. The most substantial suppression of S.I was observed in the treatment supplemented with both NCM and AV (T5), underscoring the synergistic effect between competitive exclusion and antimicrobial pressure. In contrast, NCM inoculation without AV supplementation exhibited limited efficacy, suggesting that environmental conditions and indigenous microbial communities play a pivotal role in pathogen dynamics. Genomic analysis of S.I revealed the presence of multiple resistance determinants – including *bla*CTX-M-65, efflux pump systems (MFS, RND, SMR, and ABC), and 3,278 virulence-associated genes – confirming its MDR and virulent phenotype. Strengths of this study include its integrated approach, combining *in vitro* antagonism assays, *in vivo* litter microcosms, whole-genome sequ-encing, and physicochemical analyses to evaluate microbial interactions and pathogen suppression. The use of microcosms provided a controlled yet realistic simulation of poultry production environments.

Limitations of the study include the use of a single inoculum concentration for NCM, the exclusion of microbial consortia or strain combinations with broader ecological functionality, and the focus on a specific production setting in a single geographic region. These constraints may limit the generalizability of the findings across diverse poultry systems.

Future research should explore the efficacy of multi-strain microbial consortia under varying environmental and nutritional conditions, assess long-term pathogen suppression in reused litter, and evaluate field-level outcomes across commercial farms. In addition, studies should examine the potential of integrating NCM with other sustainable biosecurity measures to mitigate antimicrobial resistance dissemination while maintaining poultry health and productivity.

## AUTHORS’ CONTRIBUTIONS

MAOB and MVL: Designed and executed the experiment. MAOB: Collected the data and performed the experimental work. MAOB, MVL, and LMP: Analyzed the data and drafted the manuscript. All authors have read and approved the final manuscript.
